# Integration of isothermal amplification with quantum dot-based fluorescence resonance energy transfer for simultaneous detection of multiple microRNAs†
†Electronic supplementary information (ESI) available. See DOI: 10.1039/c8sc00832a


**DOI:** 10.1039/c8sc00832a

**Published:** 2018-04-12

**Authors:** Juan Hu, Ming-hao Liu, Chun-yang Zhang

**Affiliations:** a College of Chemistry, Chemical Engineering and Materials Science , Collaborative Innovation Center of Functionalized Probes for Chemical Imaging in Universities of Shandong , Key Laboratory of Molecular and Nano Probes , Ministry of Education , Shandong Provincial Key Laboratory of Clean Production of Fine Chemicals , Shandong Normal University , Jinan 250014 , China . Email: cyzhang@sdnu.edu.cn ; Fax: +86 531 82615258 ; Tel: +86 531 86186033

## Abstract

MicroRNAs (miRNAs) are small non-coding RNAs that regulate important physiological processes, and their dysregulation is associated with various human diseases. Simultaneous sensitive detection of multiple miRNAs may facilitate early clinical diagnosis. In this research, we demonstrate for the first time the integration of hyperbranched rolling circle amplification (HRCA) with quantum dot (QD)-based fluorescence resonance energy transfer (FRET) for the simultaneous detection of multiple microRNAs with a single-color QD as the donor and two fluorescent dyes as the acceptors. We used miR-21 and miR-221 as target miRNAs. We designed two circular templates which may specifically hybridize with miR-21 and miR-221, respectively, for the initiation of the HRCA reaction. The products of the HRCA reaction may hybridize with both capture probes and reporter probes to form the biotinylated acceptor-labeled sandwich hybrids. The resultant sandwich hybrids can assemble on the surface of the QD, enabling efficient FRET between the QD and the acceptors, with the Cy3 signal indicating the presence of miR-21 and the Texas Red signal indicating the presence of miR-221. This assay has significant advantages of simplicity and low cost. The HRCA reaction can be performed under isothermal conditions with the same reverse primer for different target miRNAs, and the products of the HRCA reaction for both miR-21 and miR-221 can specifically hybridize with the same capture probes. This assay exhibits excellent specificity and high sensitivity with a detection limit of 7.2 × 10^–16^ M for miR-21 and 1.6 × 10^–17^ M for miR-221, and it can be used for simultaneous detection of multiple miRNAs in human cancer cells, holding great potential in biomedical research and clinical diagnosis.

## Abstract 2

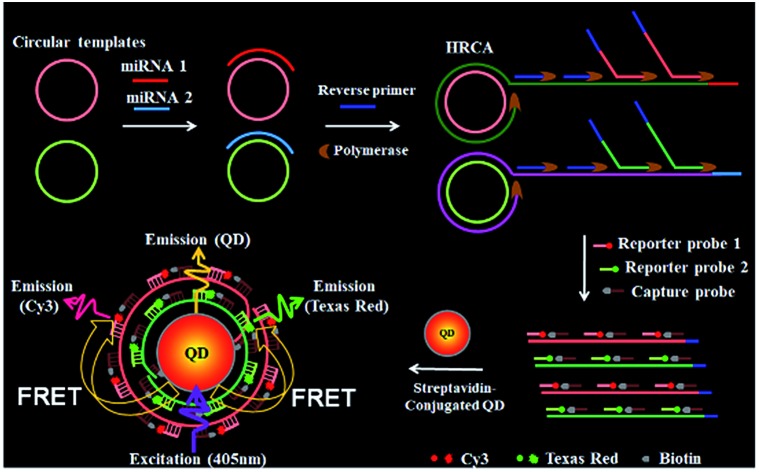
The integration of quantum dot-based fluorescence resonance energy transfer with rolling circle amplification enables simultaneous sensitive detection of multiple microRNAs.

## Introduction

MicroRNAs (miRNAs) are small non-coding endogenous RNAs, and they may repress the translation of messenger RNAs (mRNAs) and degrade target mRNAs.^1^,^2^ To date, over 1000 miRNAs have been identified in humans, which can target >30% of the human genome.^3^,^4^ They play essential roles in a variety of physiological processes including cell development and differentiation, proliferation, apoptosis and metabolism, morphogenesis and hematopoiesis.^3^,^4^ The alteration of the miRNA expression profile is a common characteristic of human tumors.^5^ Moreover, some specific miRNAs may function as oncogenes or tumour suppressors.^6^ Consequently, miRNAs have become potential biomarkers with both diagnostic and prognostic values.^2^,^3^,^5^–^7^ Due to their unique characteristics of small size, short survival time *in vitro*, high similarity between different individuals in the same family and low abundance in human total RNAs, the sensitive detection of miRNAs has remained a great challenge.^8^

The conventional methods for miRNA assay include the quantitative reverse-transcriptase polymerase chain reaction (qRT-PCR), northern blotting, microarrays, and next-generation sequencing.^8^–^10^ The qRT-PCR is a gold standard with high sensitivity, good specificity and wide dynamic range, and it can be used for absolute quantification, but it involves a sophisticated and expensive probe design (*e.g.*, a double dye-labeled probe (Taqman) and locked nucleic acid (LNA)), high-precision thermal cycling, and thermostable DNA polymerases.^8^,^11^,^12^ Northern blotting exhibits good performance for miRNA assay, but it needs large amounts of miRNA samples with poor sensitivity.^9^ Microarrays have distinct advantages of low cost and high throughput, but they suffer from poor sensitivity and involve complex data analysis.^8^ Next-generation sequencing enables quantitative evaluation of miRNA expression, but it involves substantial computational support and lacks the capability of absolute quantification.^8^,^10^ Recently, some new approaches have been developed with the involvement of bioluminescence,^13^ molecular beacons,^14^ electrochemistry,^15^ Raman spectroscopy,^16^ encoded hydrogel microparticles,^17^–^19^ encoded light-up sensors with a stem-loop-shaped miR-responsive motif,^20^ and triplexed terbium-to-QD FRET-based time-gated photoluminescence.^21^,^22^ However, their practical applications are limited due to the short bioluminescence time,^13^ the synthesis of costly fluorescent-labeled probes,^14^,^20^ the detection of unstable electrochemical signals,^15^ the requirement of separation for the elimination of interference from the background,^16^ the involvement of complicated microparticle synthesis^17^–^19^ and sophisticated spectral crosstalk correction.^21^,^22^ To improve the detection sensitivity, a series of isothermal amplification strategies^23^ have been introduced, such as rolling circle amplification (RCA),^11^,^24^,^25^ exponential amplification reaction (EXPAR),^26^ strand displacement amplification (SDA),^27^ and hairpin-mediated quadratic isothermal amplification.^28^ Among them, isothermal RCA employs short miRNAs as the templates to ligate the padlock probes,^11^,^29^ enabling homogeneously sensitive detection of miRNAs with the capability of discriminating single nucleotide difference.^11^,^12^,^24^,^25^ Despite the high amplification efficiency, these methods are limited to the detection of only a single type of miRNA due to the use of a single fluorophore,^11^,^24^ and their applications for multiplexed assay need extra templates and specially designed probes.^25^,^28^ Therefore, simultaneous and sensitive detection of multiple miRNAs still remains a great challenge.

Due to their distinct characteristics of size-tunable spectra, high quantum yield and good photostability over organic dyes, semiconductor quantum dots (QDs) have been widely used as fluorophores,^30^–^35^ fluorescence resonance energy transfer (FRET) donors^36^,^37^ and acceptors^22^ in place of organic dyes, and even as donors and acceptors simultaneously,^38^ and they have found wide applications in genomic analysis, immunoassay, fluorescence imaging and drug delivery.^39^,^40^ Especially, the QDs may function as FRET donors for homogeneous detection of small molecules, nucleic acids, proteins and cancer cells.^41^–^45^ To improve the capability of multiplexed assay, the QDs have been used for the fabrication of multiplex FRET configurations. Some typical examples include the combination of two QD donors with two dye acceptors, the integration of three QD donors with three dye acceptors,^46^–^48^ the interaction of multiple QD donors with one dye acceptor,^49^ multi-FRET distribution systems made of a central QD surrounded by pendant dyes in a sequential order,^50^–^53^ and concentric FRET configurations with multiple dye acceptors arranged symmetrically around a central QD.^54^–^56^ These multiplex FRET configurations give rise to the possibilities of multiple energy transfer between the QDs and the dyes along with dye-to-dye steps.^39^ The linear and concentric configurations enable energy transfer from a QD donor to the first dye and then to the second dye.^51^–^56^ In addition, the configuration of a single QD donor with two acceptors may transfer energy independently.^57^

Herein, we demonstrate the integration of hyperbranched rolling circle amplification (HRCA) with QD-based FRET for simultaneous detection of multiple microRNAs. We use miR-21 and miR-221 as model miRNAs, which are expressed in a wide variety of cancers and may function as oncogenes.^2^,^5^,^13^,^19^ The HRCA reaction can be performed under isothermal conditions with the same reverse primer for both miR-21 and miR-221. The products of the HRCA reaction are large amounts of single-stranded DNAs with various lengths,^11^,^24^,^25^ which can bind multiple capture probes and acceptor-labeled reporter probes. The subsequent addition of the streptavidin-coated 525QD enables the formation of 525QD–DNA–acceptor nanostructures *via* streptavidin–biotin interactions, enabling efficient FRET between the 525QD and the acceptors. We examined the case where a single QD donor interacts with multiple acceptors, and we demonstrate the use of a single QD as the energy donor in conjunction with two acceptors for simultaneous detection of endogenous miR-21 and miR-221 in different cell lines.

## Results and discussion

**Scheme 1:**
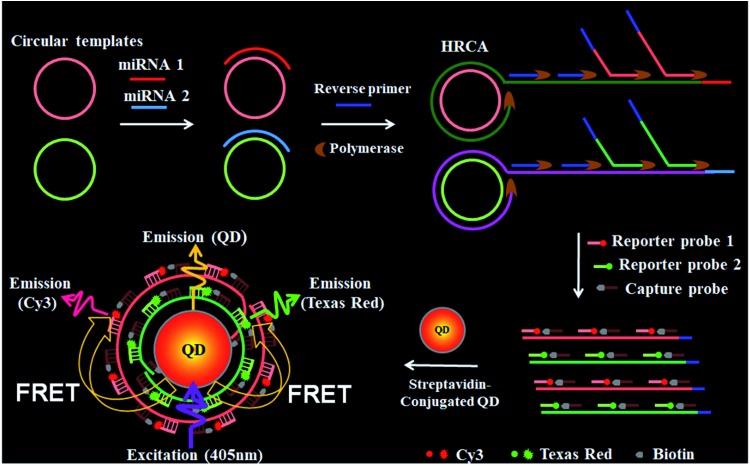
Schematic illustration of the integration of HRCA with the QD-based FRET for simultaneous detection of miR-21 and miR-221. This assay involves four steps: (1) the hybridization of miRNAs with circular templates and the subsequent HRCA reaction, (2) the hybridization of HRCA products with capture probes and reporter probes, (3) the formation of the 525QD–DNA–dye nanostructures through streptavidin–biotin binding, (4) fluorescence emission from the acceptors as a result of FRET between the 525QD and the acceptors at an excitation wavelength of 405 nm.

Scheme 1 shows the principle of HRCA-based FRET for simultaneous detection of multiple miRNAs. The 525QD, Cy3 and Texas Red are involved in this assay for the construction of a FRET-based nanosensor (Scheme 1). In this research, we used streptavidin-functionalized CdSe/ZnS core/shell QDs (525QDs) with a core size of 4 nm and an overall size of ∼15 nm after coating with a polymer shell and further conjugation with streptavidin. The 525QD has a maximum fluorescence emission at 529 nm at an excitation wavelength of 405 nm, and it may act as an excellent energy donor for both Cy3 and Texas Red due to significant spectral overlaps between the emission spectrum of the 525QD and the absorption spectra of Cy3 and Texas Red (Fig. S1, ESI†). Moreover, there is negligible excitation of either Cy3 or Texas Red at the excitation wavelength of 405 nm. In addition, the 525QD has a high quantum yield (∼0.7); Cy3 and Texas Red have high extinction coefficients (∼150 000 M^–1^ cm^–1^ for Cy3, and ∼107 000 M^–1^ cm^–1^ for Texas Red) (Table S1, ESI†). A single 525QD may couple to multiple Cy3-labeled and Texas Red-labeled sandwich hybrids (up to ∼15–30 sandwich hybrids on the basis of 5–10 streptavidins on the surface of each 525QD and 3 available biotin-binding sites per streptavidin after its conjugation to the 525QD). Therefore, the 525QD may function as both a FRET energy donor and a concentrator for signal amplification, enabling efficient FRET between the 525QD and the dye acceptor within an efficient distance of 2*R*_0_.^39^,^49^,^58^ We calculated the critical Förster distances (*R*_0_) between a streptavidin-functionalized 525QD donor and two types of acceptors (Fig. S2, ESI†). The core 525QD has a radius of 20 Å, and the radius of the streptavidin-functionalized 525QD is ∼75 Å. The Förster distance *R*_0_ is calculated on the basis of eqn (1):^39^,^43^
1
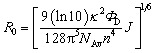
where *Φ*_D_ is the quantum yield of the streptavidin-coated 525QD donor (*Φ*_D_ = 0.7), *n* is the solvent refractive index (*n* = 1.4 for biomolecules in aqueous solution), and *κ*^2^ is the dipole orientation factor. A random temporal dipole orientation is often assumed for FRET systems with *κ*^2^ = 2/3, which is dynamic averaging for fast isotropic rotation of the donor and acceptor in ensemble measurements^39^ and is appropriate for random dipole orientations in self-assembled configurations.^50^–^53^,^57^,^58^ The spectral overlap integral *J* defined in terms of wavelength *λ* is calculated on the basis of eqn (2):^39^,^43^
2
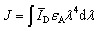
where *Ī*_D_ is the normalized emission intensity and *ε*_A_ is the wavelength-dependent extinction coefficient. Notably, when the unit of wavelength *λ* is nm and the unit of extinction coefficient is M^–1^ cm^–1^ (*ε*_A_ (570) = 150 000 M^–1^ cm^–1^ for Cy3, and *ε*_A_ (613) = 107 000 M^–1^ cm^–1^ for Texas Red), the unit of *R*_0_ = 0.02108(*κ*^2^*Φ*_D_*n*^–4^*J*)^1/6^ is nm. The Förster distance (*R*_0_) is estimated to be 67 Å for the 525QD/Cy3 pair and 54 Å for the 525QD/Texas Red pair, respectively (Table S2, ESI†).

We further calculated the separation distance between the 525QD donor and the acceptor in the FRET-based nanosensor. The separation distance (*r*) is the summation of the radius of the streptavidin-functionalized 525QD and effective width of multilayer dsDNA without taking into account the contribution of biotin and the acceptor size. The effective width (*r*_1_) of multilayer dsDNA along the radius axis (Scheme 1 and Fig. S2, ESI†) can be estimated on the basis of eqn (3):

3 *r*_1_ = 2*H* + (*H* – 1)*g*

where *H* is the number of double-helical domains along the axis, and *g* is the interhelical gap size between cross-overs along the same axis.^59^ Based on the assumption that the unhydrated helical diameter of dsDNA is 20 Å and an interhelical gap produced by electrostatic repulsion is 6 Å under the buffer conditions,^60^ the change of *H* from 1 to 2 may result in the increase of effective width from 20 Å to 46 Å. When the layer number of dsDNA assembled on the surface of the 525QD is 2 for the 525QD–Cy3-based nanosensor and 1 for the 525QD–Texas Red-based nanosensor, the separation distance is estimated to be 121 Å for the 525QD/Cy3 pair and 95 Å for the 525QD/Texas Red pair, within the efficient distance of 2*R*_0_ (2*R*_0_ = 134 Å for the 525QD/Cy3 pair, and 2*R*_0_ = 108 Å for the 525QD/Texas Red pair). Taking into account the fact that the DNA double-helical domain has a natural helicity of 32 bp per full 3 turn (33.75° per bp average twist),^60^ the separation distance is 127–147 Å for the 525QD/Cy3 pair and 101–121 Å for the 525QD/Texas Red pair when the layer number of dsDNA assembled on the surface of the 525QD is 3 for the 525QD–Cy3-based nanosensor and 2 for the 525QD–Texas Red-based nanosensor, still within the efficient distance of 2*R*_0_. Despite no much difference between the two nanostructures, FRET can only occur within the efficient donor–acceptor distance of 2*R*_0_,^39^ leading to different measurable distances (*i.e.*, 134 Å for the 525QD/Cy3 pair and 108 Å for the 525QD/Texas Red pair) and different predicted layers (*i.e.*, 1–3 layers for the 525QD–Cy3-based nanosensor and 1–2 for the 525QD–Texas Red-based nanosensor). In addition, there are negligible stacked homo- and hetero-FRET in the 525QD–DNA–Cy3 nanostructure, the 525QD–DNA–Texas Red nanostructure, and the 525QD–DNA–Cy3/Texas Red nanostructure (Fig. S3, ESI†). Consequently, the 525QD is the only donor in this multiple FRET system without the involvement of either two different QD donors for two different dye acceptors or three QD donors for three dye acceptors.^46^–^48^

In this FRET-based nanosensor, a single QD may interact with multiple acceptors, significantly improving the FRET efficiency. The FRET efficiency (*E*) may be calculated on the basis of eqn (4):^39^4
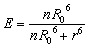
where *n* is the average number of acceptor molecules interacting with one donor, *R*_0_ is the calculated Förster distance, and *r* is the average donor–acceptor separation distance. We further calculated theoretically the number of acceptors on the surface of a single 525QD based on the assumption that multilayer dsDNA is assembled on the surface of the 525QD (Scheme 1 and Fig. S2, ESI†). Every repeat of HRCA is 70–71 bp (Table 1), and the capacity of one repeat is calculated to be ∼74 732 Å^3^ for the 525QD/Cy3 pair and ∼75 674 Å^3^ for the 525QD/Texas Red pair on the basis of the assumption that the unhydrated diameter of dsDNA is 20 Å.^60^ For the 525QD–DNA–Cy3 nanostructure, on the basis of the assumption that the efficient distance of 2*R*_0_ is 134 Å for the 525QD/Cy3 pair and the radius of the streptavidin-functionalized 525QD is ∼75 Å, the surface capacity of the 525QD with multilayer dsDNA is calculated to be 8 307 305 Å^3^, and the maximum number of repeats assembled on the surface of a single 525QD is estimated to be 111, (*i.e.*, a maximum of 111 Cy3 acceptors may be assembled on the surface of a single 525QD). For the 525QD–DNA–Texas Red nanostructure, on the basis of the assumption that the efficient distance of 2*R*_0_ is 108 Å for the 525QD/Texas Red pair and the radius of the streptavidin-functionalized 525QD is ∼75 Å, the surface capacity of the 525QD with multilayer dsDNA is calculated to be 3 507 744 Å^3^, and the maximum number of repeats assembled on the surface of a single 525QD is estimated to be 46, (*i.e.*, a maximum of 46 Texas Red acceptors may be assembled on the surface of a single 525QD), leading to improved FRET efficiency in this FRET-based nanosensor.

**Table 1:** Sequences of the oligonucleotides^*a*^

Note	Sequence (5′–3′)
miR-21	UAG CUU AUC AGA CUG AUG UUG A
miR-221	AGC UAC AUU GUC UGC UGG GUU UC
Circular template-21	CAG AAC AGC ACA AGA CAG GAC AAG ACA CAC GCC GAA **TCA ACA TCA GTC TGA TAA GCT A**CC AGA CAG ACG A
Circular template-221	CAG AAC AGC ACA AGA CAG GAC AAG ACA CAC GCC GAA **GAA ACC CAG CAG ACA ATG TAG CT**C CAG ACA GAC GA
Reverse primer	GAC AGA CGA CAG AAC AG
Capture probe	Biotin – GGC GTG TGT CTT GTC CTG
Reporter probe-21	TAG CTT ATC AGA CTG ATG TTG A – Cy3
Reporter probe-221	AGC TAC ATT GTC TGC TGG GTT TC – Texas Red
Let-7a	UGA GGU AGU AGG UUG UAU AGU U
RNA-1	UAG CUU AUC ACA CUG AUG UUG A
RNA-2	AGC UAC AUU GUC GGC UGG GUU UC

^*a*^In the circular template, the hybridization region for target miRNA is shown in bold. The underlined characters of RNA-1 represent the different base compared with target miR-21. Underlined characters of RNA-2 represent the different base compared with target miR-221.

As a proof of concept, we used miRNA-21 (miR-21) and miRNA-221 (miR-221) as model miRNAs and designed two circular templates for miR-21 and miR-221, respectively, and one reverse primer for HRCA (Table 1). MiR-21 is overexpressed in a variety of cancers including breast, ovary, cervix, colon, lung, liver, brain, esophagus, prostate, and thyroid cancers.^2^,^5^ MiR-221 is overexpressed in glioblastomas, prostate, pancreatic, hepatocellular, and thyroid cancers.^5^ The 70-nt circular template for miR-21 (circular template-21) contains a target-complementary sequence and two special regions for HRCA. miR-21 may specifically bind to the complementary region of circular template-21 to initiate the HRCA reaction in the presence of Bst DNA polymerase, dTNPs and the reverse primer, producing a long DNA strand with repeated sequences. Notably, HRCA is an isothermally exponential amplification,^11^ and it produces large amounts of single-stranded DNAs with various lengths, which can hybridize with the same capture probes without the need for the synthesis of specific capture probes for different target miRNAs.^26^ The HRCA products may hybridize with the biotinylated capture probes and Cy3-labeled reporter probes to form sandwich hybrids. The resultant sandwich hybrids can assemble on the surface of the 525QD to obtain the 525QD–DNA–Cy3 nanostructure through specific biotin–streptavidin interactions. When the nanostructures are excited at a wavelength of 405 nm, the fluorescence signals of both the 525QD and Cy3 can be observed simultaneously due to FRET from the 525QD to Cy3 (Scheme 1). Similar approach may be applied for the detection of miR-221 using the circular templates for miR-221 (circular template-221), Texas Red-labeled reporter probes and the same capture probes. In addition, we investigated the wrapping of a multiple biotinylated HRCA product around the 525QD *via* streptavidin–biotin interactions by measuring the fluorescence spectra in the presence of the 525QD and 525QD + reporter probes with various amounts of the biotinylated capture probe, respectively, and we found two-layer dsDNA for the 525QD/Cy3 pair and one-layer dsDNA for the 525QD/Texas Red pair (Fig. S4 and S5, ESI†). Importantly, this approach can be used to simultaneously detect miR-21 and miR-221 using circular template-21 and circular template-221, Cy3-labeled and Texas Red-labeled reporter probes, and the same capture probes, with the Cy3 signal indicating the presence of miR-21 and the Texas Red signal indicating the presence of miR-221 (Scheme 1).

**Fig. 1:**
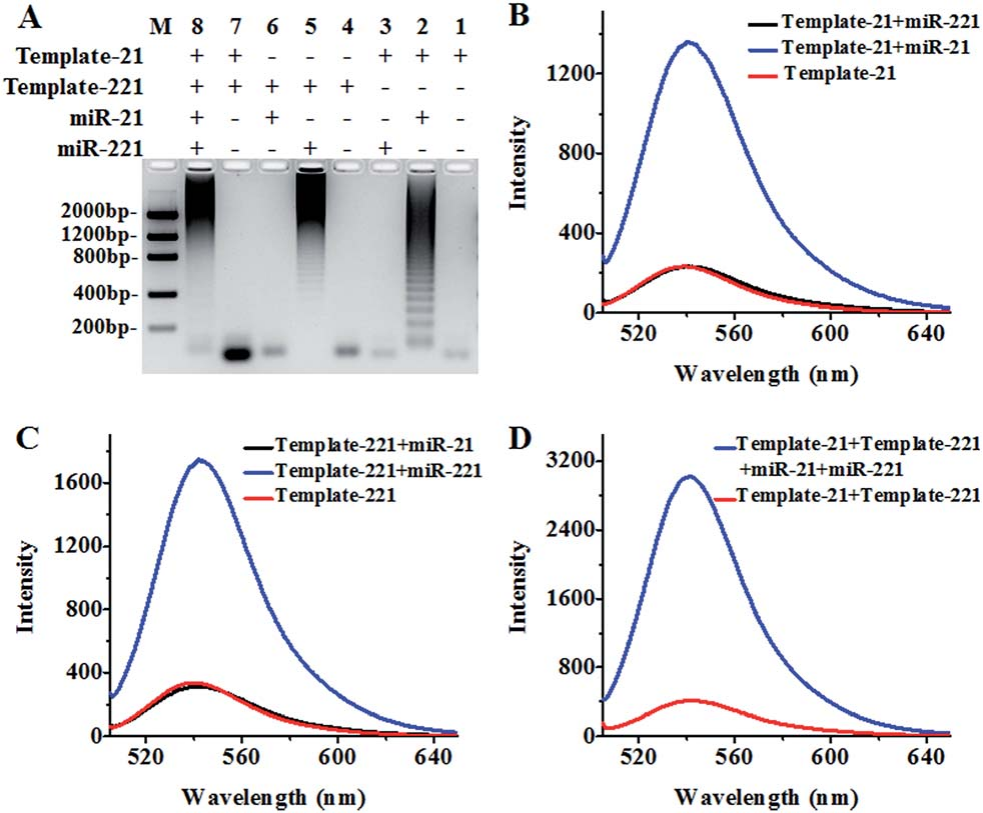
(A) Agarose gel electrophoresis analysis of the HRCA products. Lane M is the DNA ladder marker. Lanes 1–3 represent HRCA products in the presence of circular template-21 (lane 1), circular template-21 + miR-21 (lane 2) and circular template-21 + miR-221 (lane 3). Lanes 4–6 represent the HRCA products in the presence of circular template-221 (lane 4), circular template-221 + miR-221 (lane 5) and circular template-221 + miR-21 (lane 6). Lanes 7 and 8 represent the HRCA products in the presence of circular template-21 + circular template-221 (lane 7) and circular template-21 + miR-21 + circular template-221 + miR-221 (lane 8). (B) Fluorescence measurement of the HRCA products with SYBR Gold as the fluorescent indicator in the presence of circular template-21 (red line), circular template-21 + miR-21 (blue line) and circular template-21 + miR-221 (black line). (C) Fluorescence measurement of the HRCA products with SYBR Gold as the fluorescent indicator in the presence of circular template-221 (red line), circular template-221 + miR-221 (blue line) and circular template-221 + miR-21 (black line). (D) Fluorescence measurement of the HRCA products with SYBR Gold as the fluorescent indicator in the presence of circular template-21 + circular template-221 (red line) and circular template-21 + miR-21 + circular template-221 + miR-221 (blue line). The miR-21 concentration is 0.2 nM, the miR-221 concentration is 0.2 nM, the circular template-21 concentration is 10 nM and the circular template-221 concentration is 10 nM.

We used agarose gel electrophoresis and fluorescence measurements to verify the products of the HRCA reaction (Fig. 1). There is only one band of circular template when miR-21 and miR-221 are absent (Fig. 1A, lanes 1, 4 and 7), indicating no occurrence of the HRCA reaction. In contrast, distinct bands with different molecular weights are observed in the presence of miR-21 and circular template-21 (Fig. 1A, lane 2), indicating the occurrence of miR-21-initiated HRCA. Similar bands with different molecular weights are observed in the presence of miR-221 and circular template-221 (Fig. 1A, lane 5), indicating the occurrence of miR-221-initiated HRCA. However, no new band is observed in the presence of either miR-221 + circular template-21 (Fig. 1A, lane 3) or miR-21 + circular template-221 (Fig. 1A, lane 6). In addition, distinct bands with different molecular weights are observed in the presence of miR-21, miR-221, circular template-21 and circular template-221 (Fig. 1A, lane 8).

We further measured the emission spectra of RCA products with SYBR Gold as the fluorescent indicator. SYBR Gold can stain all the involved nucleic acids. The results of fluorescence measurements (Fig. 1B–D) are consistent with those of agarose gel electrophoresis. In the presence of only circular templates and a reverse primer, a low fluorescence signal (Fig. 1B–D, red line) is observed as a result of the staining of circular templates and the reverse primer by SYBR Gold (note: since the same reverse primer is required for all HRCA reactions, the reverse primer is not indicated in Fig. 1). In contrast, an enhanced fluorescence signal is detected in the presence of circular template-21 and miR-21 (Fig. 1B, blue line). Similarly, an enhanced fluorescence signal is detected in the presence of circular template-221 and miR-221 (Fig. 1C, blue line). However, no enhanced fluorescence signal is observed in the presence of circular template-21 + miR-221 (Fig. 1B, black line) and circular template-221 + miR-21 (Fig. 1C, black line). These results demonstrate that the HRCA reaction can only be triggered by target miRNAs in the presence of a specific circular template. Notably, the fluorescence signal with the involvement of HRCA is extremely higher than that without the involvement of HRCA in response to the same amount of target miRNA (Fig. S6, ESI†). A near zero fluorescence signal is obtained without the involvement of HRCA despite the existence of the same amount of target miRNA. In theory, fold amplification may be calculated by dividing the number of tandem repeats by the input miRNA.^61^ Fold amplification is calculated to be ∼1215 for miR-21-triggered HRCA and ∼1507 for miR-221-triggered HRCA (Fig. S6, ESI†). As expected, an enhanced fluorescence signal is detected in the presence of circular template-21, miR-21, circular template-221 and miR-221 (Fig. 1D, blue line), whose fluorescence intensity is the summation of fluorescence intensities in response to circular template-21 + miR-21 (Fig. 1B, blue line) and circular template-221 + miR-221 (Fig. 1C, blue line), suggesting that the two circular templates used in this research do not interfere with each other and they can be used for simultaneous detection of multiple miRNAs.

**Fig. 2:**
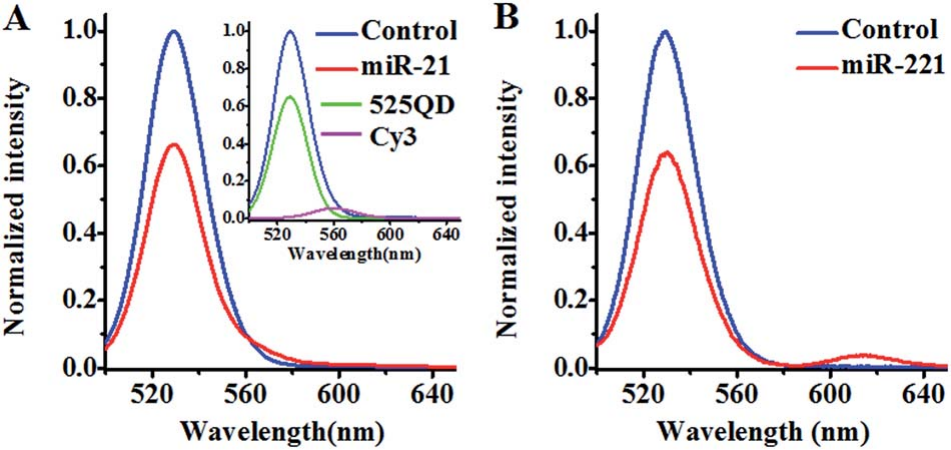
(A) Measurements of the 525QD and Cy3 emission spectra in the absence (control, blue line) and presence of miR-21 (red line). The individual contribution of the 525QD is shown in green, and the individual contribution of Cy3 is shown in magenta (inset of (A)). (B) Measurements of the 525QD and Texas Red emission spectra in the absence (control, blue line) and presence of miR-221 (red line).

We investigated the FRET between the 525QD and Cy3 (Fig. 2A) induced by miR-21-initiated HRCA. No Cy3 signal is observed in the absence of miR-21 (Fig. 2A, blue line), indicating no occurrence of FRET between the 525QD and Cy3 because no Cy3-labeled reporter probe can be assembled on the 525QD without miR-21-initiated HRCA. In contrast, the presence of miR-21 induces the decrease of 525QD fluorescence intensity accompanied by the increase of Cy3 fluorescence intensity (Fig. 2A, red line), indicating the occurrence of FRET from the 525QD to Cy3 as a result of the assembly of the Cy3-labeled reporter probe on the 525QD. We further analyzed the individual contributions of 525QD and Cy3 to the composite spectrum (Fig. S7, ESI†),^49^,^57^ with the individual contribution of 525QD and Cy3 being shown in green and magenta, respectively (inset of Fig. 2A). The FRET efficiency is calculated to be 33.7% for the 525QD/Cy3 pair on the basis of eqn (5).^39^,^43^
5
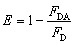
where *F*_D_ and *F*_DA_ are the fluorescence integrated intensities of the QD donors in the absence and presence of acceptors, respectively.

We investigated the FRET between 525QD and Texas Red induced by miR-221-initiated HRCA as well (Fig. 2B). No Texas Red signal is observed in the absence of miR-221 (Fig. 2B, blue line), indicating no occurrence of FRET between the 525QD and Texas Red because no Texas Red-labeled reporter probe can be assembled on 525QD without miR-221-initiated HRCA. In contrast, the presence of miR-221 results in the decrease of 525QD fluorescence intensity and the increase of Texas Red fluorescence intensity (Fig. 2B, red line). The FRET efficiency is calculated to be 35.8% for the 525QD/Texas Red pair on the basis of eqn (5).^39^,^43^ These results clearly demonstrate that target microRNA-initiated HRCA may induce efficient FRET for both the 525QD/Cy3 pair and 525QD/Texas Red pair. Furthermore, we measured the number of acceptors per 525QD based on the variation of FRET efficiency with the reporter probe-to-525QD ratio (the molar ratio of biotinylated capture probe to Cy3-labeled reporter probe is kept at 1 : 1). As shown in Fig. S8 (ESI),† the FRET efficiency improves with the increasing reporter probe-to-525QD ratio and reaches a plateau at the ratio of 15 : 1, suggesting that a maximum of 15 Cy3-labeled reporter probes may be assembled on the surface of a single QD and make a contribution to the FRET efficiency experimentally.^62^ The value of 15 acceptors is different from the theoretically calculated maximum number of 46–111 acceptors per QD in the case of multilayer dsDNA assembled on the surface of a single 525QD. The difference may be explained by the following two reasons: (1) some acceptors might be located beyond the efficient range of 2*R*_0_, and (2) some acceptor-labeled sandwich hybrids might not be assembled on the surface of the 525QD due to the steric hindrance. Notably, when *n* is 15, the average donor–acceptor separation distance was calculated to be 118 Å for the 525QD/Cy3 pair and 93 Å for the 525QD/Texas Red pair on the basis of eqn (4),^39^ within the efficient range of FRET (2*R*_0_ = 134 Å for the 525QD/Cy3 pair and 2*R*_0_ = 108 Å for the 525QD/Texas Red pair). Interestingly, these values are consistent with the theoretically calculated separation distance of 121 Å (two-layer dsDNA) for the 525QD/Cy3 pair and 95 Å (one-layer dsDNA) for the 525QD/Texas Red pair.

**Fig. 3:**
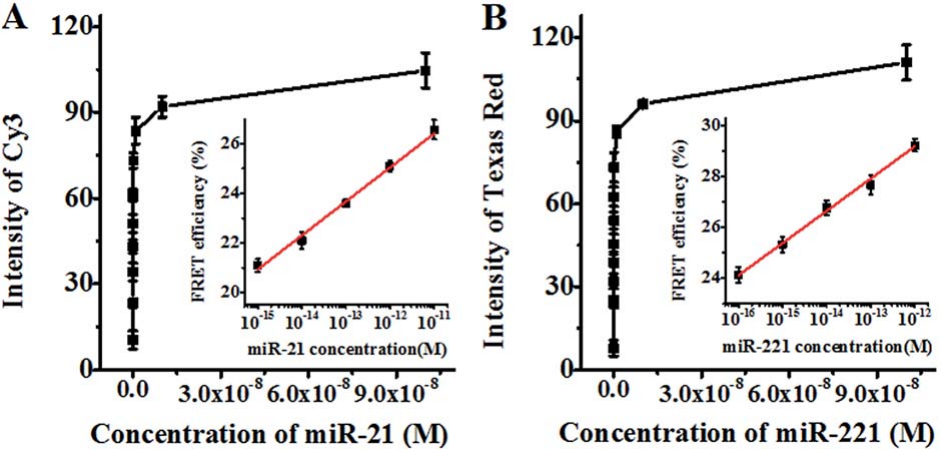
(A) Variation of Cy3 fluorescence intensity with the concentration of miR-21. The inset shows the log-linear correlation between FRET efficiency and the concentration of miR-21. (B) Variation of Texas Red fluorescence intensity with the concentration of miR-221. The inset shows the log-linear correlation between FRET efficiency and the concentration of miR-221. The concentration of streptavidin-coated 525QDs is 5 nM for all experiments. Error bars show the standard deviation of three experiments.

We further investigated the sensitivity of the proposed method for miR-21 and miR-221 assays under the optimised experimental conditions (Fig. S9, ESI†). As shown in Fig. 3A, the fluorescence intensity of Cy3 improves as a function of miR-21 concentration in the range from 0 to 1 × 10^–7^ M. In the logarithmic scale, the FRET efficiency shows a linear correlation with the miR-21 concentration in the range from 1 × 10^–15^ M to 1 × 10^–11^ M (inset of Fig. 3A). The correlation equation is *E* = 41.48 + 1.37 log_10_ *C* (*R*^2^ = 0.990), where *E* and *C* are the FRET efficiency and the concentration of miR-21 (M), respectively. The detection limit is calculated to be 7.2 × 10^–16^ M on the basis of the average signal of blank plus three times the standard deviation. As shown in Fig. 3B, the fluorescence intensity of Texas Red improves with the increasing concentration of miR-221 from 0 to 1 × 10^–7^ M. In the logarithmic scale, the FRET efficiency shows a linear correlation with the miR-221 concentration in the range from 1 × 10^–16^ M to 1 × 10^–12^ M (inset of Fig. 3B). The correlation equation is *E* = 44.38 + 1.27 log_10_ *C* (*R*^2^ = 0.993), where *E* and *C* are the FRET efficiency and the concentration of miR-221 (M), respectively. The detection limit is calculated to be 1.6 × 10^–17^ M based on the average signal of blank plus three times the standard deviation. Notably, the sensitivity of the proposed method has improved by as much as 7 orders of magnitude as compared with that of the immobilized QD-based FRET assay (10 nM),^57^ and more than 5 orders of magnitude as compared with that of the quenched Staudinger-triggered (Q-STAR) probe-based RCA assay (0.2 nM),^63^ and more than 2 orders of magnitude as compared with that of the fluorescently labeled peptide nucleic acid and graphene oxide-based RCA assay (0.4 pM),^64^ and more than 1 order of magnitude as compared with that of the gold–silver nanomushroom probe-based SERS assay (10 fM).^16^ The improved sensitivity may be attributed to (1) the specific hybridization of the circular templates with target miRNAs, (2) the exponential amplification capability of HRCA, (3) the efficient FRET between the 525QD and the acceptor due to the formation of the 525QD–DNA–acceptor nanostructure, and (4) the improved FRET efficiency resulting from the interaction of a single QD donor with multiple acceptors.

**Fig. 4:**
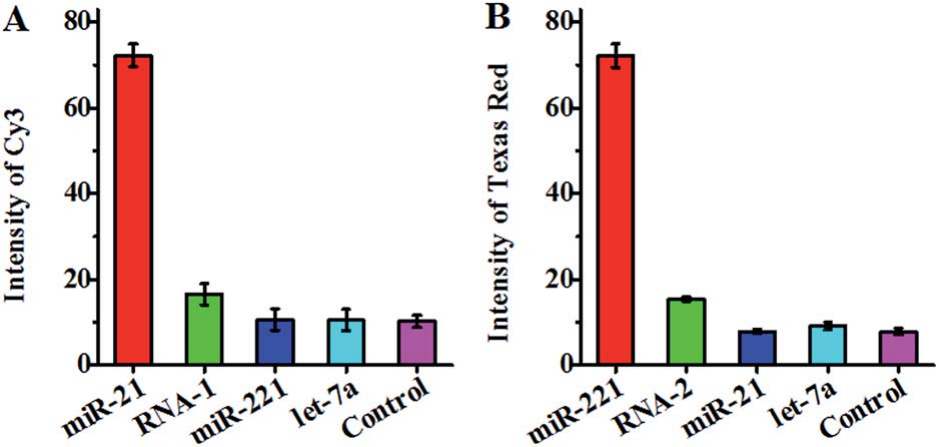
(A) Measurement of the Cy3 fluorescence signal in response to miR-21 (red column), single-base mismatched RNA (RNA-1, green column), miR-221 (blue column), let-7a (cyan column), and the control without any miRNA (magenta column) using circular template-21. (B) Measurement of the Texas Red fluorescence signal in response to miR-221 (red column), single-base mismatched RNA (RNA-2, green column), miR-21 (blue column), let-7a (cyan column), and the control without any miRNA (magenta column) using circular template-221. The concentration of each miR-21, miR-221, RNA-1, RNA-2, and let-7a is 0.1 nM. Error bars show the standard deviation of three experiments.

To investigate the specificity of the proposed method, we measured target miRNA, single-base mismatched RNA (*i.e.*, RNA-1 and RNA-2) and noncomplementary miRNA (*i.e.*, let-7a) (Table 1) at the same concentration using circular template-21 and circular template-221. In the presence of circular template-21, a distinct Cy3 fluorescence signal is observed in response to miR-21 instead of RNA-1, miR-221 and let-7a (Fig. 4A). Notably, the Cy3 fluorescence signal in response to target miR-21 is 7.1-fold higher than that in response to the control without any miRNA, 6.8–6.9-fold higher than that in response to noncomplementary miRNA (*i.e.*, miR-221 and let-7a), and 4.4-fold higher than that in response to single-base mismatched RNA (*i.e.*, RNA-1). In the presence of circular template-221, a distinct Texas Red fluorescence signal is observed in response to miR-221 instead of RNA-2, miR-21 and let-7a (Fig. 4B). Notably, the Texas Red fluorescence signal in response to target miR-221 is 9.3-fold higher than that in response to the control without any miRNA, 7.9–9.2-fold higher than that in response to noncomplementary miRNA (*i.e.*, miR-21 and let-7a), and 4.7-fold higher than that in response to single-base mismatched RNA (*i.e.*, RNA-2). These results suggest the high specificity of the proposed method for miRNA assay with the capability of discriminating even single-nucleotide difference. The high specificity might be attributed to (1) specific hybridization of circular templates with target miRNAs, (2) specific sandwich hybridization of the HRCA products with both capture probes and reporter probes, (3) specific biotin–streptavidin interactions for the formation of 525QD–DNA–acceptor nanostructures, and (4) good spectral resolution between Cy3 emission and Texas Red emission (Fig. S1, ESI†).

**Fig. 5:**
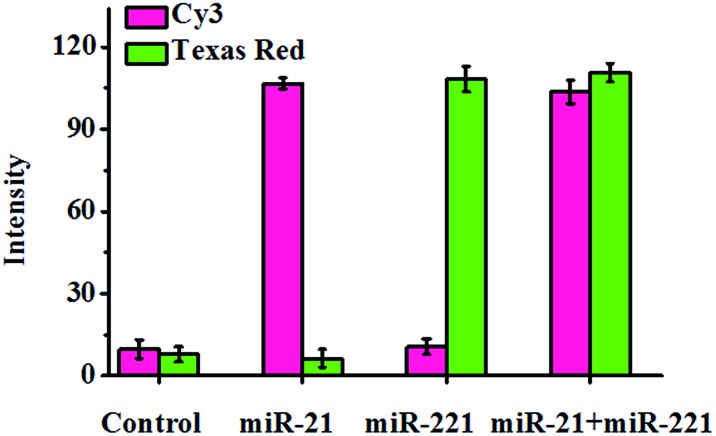
Simultaneous detection of miR-21 and miR-221. The pink column shows the fluorescence intensity of Cy3, and the green column shows the fluorescence intensity of Texas Red. The miR-21 concentration is 0.1 μM, and the miR-221 concentration is 0.1 μM. Error bars show the standard deviation of three experiments.

To investigate the capability of the proposed method for the simultaneous detection of multiple miRNAs, we measured the mixture of miR-21 and miR-221 using both circular template-21 and circular template-221 (Fig. 5). In the presence of miR-21, a distinct Cy3 fluorescence signal is observed, but no Texas Red fluorescence signal is detected. However in the presence of miR-221, a distinct Texas Red fluorescence signal is observed, but no Cy3 fluorescence signal is detected. Only the co-existence of miR-21 and miR-221 can induce distinct Cy3 and Texas Red fluorescence signals simultaneously. These results clearly demonstrate the feasibility of the proposed method for the simultaneous detection of multiple miRNAs.

**Fig. 6:**
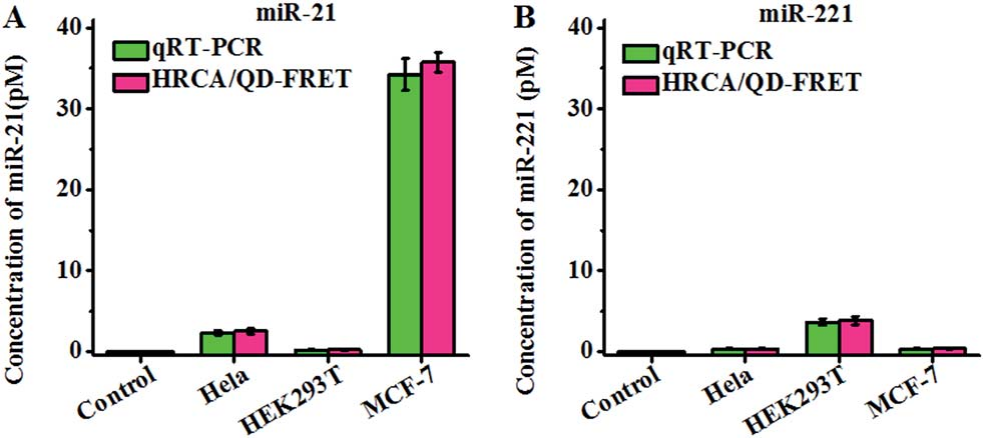
Measurements of miR-21 (A) and miR-221 (B) in cell extract samples by using qRT-PCR (green column) and the proposed method (pink column). 10 ng of total RNA extracted from MCF-7 cells, HEK293T cells and HeLa cells was measured, respectively, with 0 ng of total RNA extracted as the control group. Error bars show the standard deviations of three experiments.

Both miR-21 and miR-221 are expressed in human cancer cells.^2^,^5^,^13^,^19^ To investigate the feasibility of the proposed method for real sample analysis, we detected the endogenous miR-21 and miR-221 levels by measuring 10 ng of total RNA extracted from MCF-7 cells, HEK293T cells and HeLa cells, respectively, with 0 ng of total RNA as the control group. The concentrations of miR-21 and miR-221 are calculated according to the calibration curve in Fig. 3. The fluorescence signals of Cy3 and Texas Red obtained from MCF-7 cells are much higher than that obtained from the control group, with a higher level of miR-21 and a lower level of miR-221 in MCF-7 cells (Fig. 6), consistent with the result of the bioluminescent assay and the microarray assay.^13^,^65^ We found that miR-21 and miR-221 are expressed at varying levels in HEK293T cells and HeLa cells. The level of miR-21 is lower than that of miR-221 in HEK293T cells, consistent with previous sensor array assay.^66^ The level of miR-21 is much higher than that of miR-221 in HeLa cells, consistent with previous library screening assay.^67^ In addition, we compared our results with those obtained by qRT-PCR using the same batch of extract samples from MCF-7 cells, HEK293T cells and HeLa cells (Fig. S10 and S11, ESI†). The qRT-PCR is a standard method for miRNA profiling. Fig. 6 shows that our results are in good agreement with those obtained by qRT-PCR. These results clearly demonstrate the feasibility and accuracy of the proposed method for real sample analysis.

## Conclusions

In summary, we have demonstrated for the first time the integration of HRCA with QD-based FRET for simultaneous detection of multiple microRNAs with a single-color QD as the donor and two fluorescent dyes as the acceptors. This assay has significant advantages of simplicity and low cost. The HRCA reaction may be performed under isothermal conditions with the same reverse primer for different target miRNAs, and the products of the HRCA reaction for both miR-21 and miR-221 may specifically hybridize with the same capture probes. Moreover, the specific hybridization of circular templates with target miRNAs and the specific sandwich hybridization of HRCA products with both capture probes and reporter probes guarantee the high selectivity of the proposed method. In addition, the exponential amplification efficiency of HRCA and the improved FRET efficiency of the QD-based nanosensor ensure the high sensitivity of the proposed method. This assay shows excellent specificity and high sensitivity with a detection limit of 7.2 × 10^–16^ M for miR-21 and 1.6 × 10^–17^ M for miR-221, and it can be used for simultaneous detection of multiple miRNAs. Importantly, this method can be applied for real sample analysis and exhibits good performance in human cancer cell assay, holding great potential for further applications in biomedical research and clinical diagnostics.

## Experimental

### Materials

The HPLC-purified oligonucleotides (Table 1) were synthesized by TaKaRa Biotechnology Co., Lid. (Dalian, China). The circular template DNA was prepared from the corresponding linear templates by TaKaRa Biotechnology Co., Lid. (Dalian, China). The Bst DNA polymerase large fragment and deoxynucleotide solution mixture (dNTPs) were purchased from New England Biolabs (Ipswich, MA, USA). Streptavidin-coated 525 nm emission QDs (525QDs), SYBR Gold nucleic acid gel stain and diethyl pyrocarbonate (DEPC)-treated water were obtained from Invitrogen Co. (Carlsbad, CA, USA). A RNase inhibitor was purchased from Takara Biotechnology Co., Ltd. (Dalian, China). Human breast cancer cell line (MCF-7 cells), human embryonic kidney cell line (HEK293T cells) and human cervical cancer cells (HeLa cells) were purchased from Cell Bank of Chinese Academy of Sciences (Shanghai, China).

### HRCA reaction

Before the HRCA reaction, 10 nM circular template-21 and/or circular template-221, 100 nM reverse primer and target miRNA at a certain concentration were incubated in 1× ThermoPol reaction buffer (20 mM Tris–HCl (pH 8.8), 10 mM KCl, 10 mM (NH_4_)_2_SO_4_, 2 mM MgSO_4_ and 0.1% Triton-100) at 95 °C for 5 min. After slowly cooling to room temperature, 8 U of Bst DNA polymerase, 200 μM dNTPs and 16 U of RNase inhibitor were added to the mixture in a final volume of 20 μL. The HRCA reaction was carried out at 60 °C for 1 h, followed by heat inactivation at 80 °C for 20 min.

The HRCA reaction was monitored with 1× SYBR Gold as the fluorescent indicator. 2% agarose gel was used to analyze the products of the HRCA reaction in 1× TAE buffer (40 mM Tris–ethylic acid, 2 mM EDTA) at a constant voltage of 110 V for 50 min. The gel was scanned by using a Bio-Rad ChemiDoc imaging system (California, USA). The amplification product was mixed with SYBR Gold, and the fluorescence emission spectra were measured by using a Hitachi F-7000 fluorometer (Tokyo, Japan) at an excitation wavelength of 495 nm. The value of (*F* – *F*_0_)/*F*_0_ is used to optimize the experimental conditions, where *F* and *F*_0_ are the fluorescence intensities at 540 nm in the presence and absence of miRNA, respectively.

### Hybridization reaction

20 μL of the HRCA reaction product, 0.3 μL of 20 μM Cy3- and/or Texas Red-modified reporter probes and 0.3 μL of 20 μM biotinylated capture probes were added to a buffer solution containing 100 mM Tris–HCl (pH 8.0), 10 mM (NH_4_)_2_SO_4_, 3 mM MgCl_2_ in a final volume of 78 μL. The mixture was heated at 95 °C for 5 min, and then incubated at 45 °C for 1 h. After cooling to room temperature, 2 μL of 0.2 μM streptavidin-coated 525QDs was added to the mixture and incubated at room temperature for 15 min with a final concentration of 5 nM for the streptavidin-coated 525QDs. The fluorescence emission spectra of the hybridization products were measured by using a fluorometer at an excitation wavelength of 405 nm. The individual contributions of Cy3 and Texas Red to the composite spectra were analyzed.^49^,^57^

### MiRNA extraction and real sample analysis

MCF-7 cells, HEK293T cells and HeLa cells were cultured in RPMI 1640 medium with 10% fetal bovine serum (FBS, Invitrogen, USA) at 37 °C under 5% CO_2_. The total RNA containing miRNA were extracted by using the SanPrep Column microRNA Mini-Preps Kit (Sangon Biotech, Shanghai). The concentration of total RNA was determined by measuring the absorbance at 260 nm and 280 nm with a Nanodrop 2000C Spectrophotometer (Thermo Scientific). For qRT-PCR assay, the total RNA was quantified by using the Mir-X™ miRNA qRT-PCR SYBR Kit (TaKaRa, Dalian, China) according to the manufacturer's manual.

## Conflicts of interest

There are no conflicts to declare.

## Supplementary Material

Supplementary information
