# Supplementary material for: Integration of isothermal amplification with quantum dot-based fluorescence resonance energy transfer for simultaneous detection of multiple microRNAs
Source: Chem Sci. 2018 Apr 12;9(18):4258–67. doi: 10.1039/c8sc00832a (PMC5944210; doi:10.1039/c8sc00832a)
Supplement: Supplementary file 1 [file SC-009-C8SC00832A-s001.pdf]

Electronic Supplementary Material (ESI) for Chemical Science.
This journal is © The Royal Society of Chemistry 2018

Electronic Supplementary Information

Integration of isothermal amplification with quantum dot-based fluorescence resonance energy transfer for simultaneous detection of multiple microRNAs

Juan Hu,† Ming-hao Liu,† and Chun-yang Zhang*

College of Chemistry, Chemical Engineering and Materials Science, Collaborative Innovation Center of Functionalized Probes for Chemical Imaging in Universities of Shandong, Key Laboratory of Molecular and Nano Probes, Ministry of Education, Shandong Provincial Key Laboratory of Clean Production of Fine Chemicals, Shandong Normal University, Jinan 250014, China

* Corresponding author. Tel.: +86 0531-86186033; Fax: +86 0531-82615258. E-mail: cyzhang@sdnu.edu.cn.

Table of Contents

Normalized emission and absorption spectra of fluorophores……S3

Photophysical properties of the fluorophores……S3

Overlap integrals and the calculated Förster distances for the donor-acceptor pairs……S4

A schematic depicting the Förster distance (R_0) of an idealized streptavidin-functionalized 525QD donor and a Cy3 acceptor and a Texas Red acceptor……S5

Study homo and hetero FRET……S6

Study the absorption of Cy3- and Texas Red-labeled reporter probes……S9

Study the wrapping of multiple biotinylated HRCA product around 525QD …… S10

Enhanced signal induced by HRCA……S12

Analysis of the individual contributions of 525QD and Cy3 to the composite spectrum……S14

Variance of FRET efficiency as a function of the ratio of Cy3-labeled reporter probe to 525QD in the 525QD-Cy3 FRET system……S15

Optimization of experimental conditions……S15

Quantitative reverse-transcriptase polymerase chain reaction……S18

Measurement of miR-21 and miR-221 in MCF-7 cells, HEK293T cells and HeLa cells by qRT-PCR……S19

References……S20

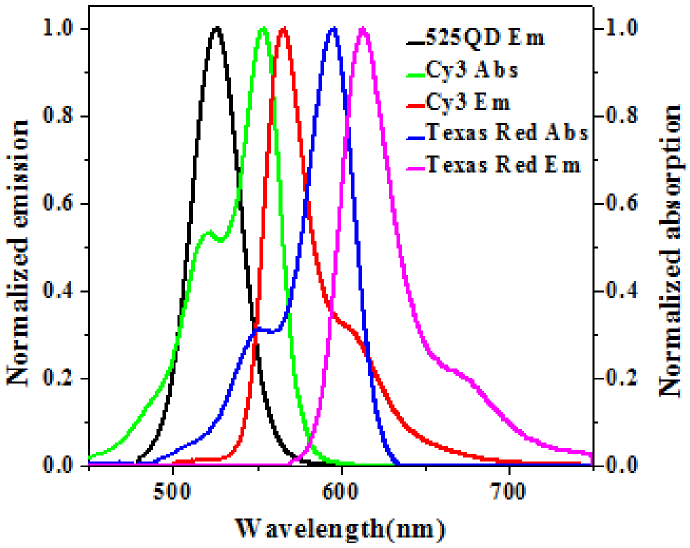

Figure S1. Normalized emission (Em) and absorption spectra (Abs) of fluorophores used in FRET with a 525QD donor and two acceptors. Black line, emission spectrum of 525QD; green line, absorption spectrum of Cy3; red line, emission spectrum of Cy3; blue line, absorption spectrum of Texas Red; magenta line, emission spectrum of Texas Red.

Table S1. Photophysical properties of the fluorophores.

fluorophore	QY	ε_{max} ($M^{-1}cm^{-1}$)	$\lambda_{max\ Abs.}$ (nm)	$\lambda_{max\ Em.}$ (nm)
525QD	0.7*	360,000 (405 nm)	405	529
Cy3	0.15	150,000	550	570
Texas Red	0.27	107,000	595	613

QY, quantum yield; ε, extinction coefficient; abs., absorption; em., emission. * is from certificates of analysis (COA).

Table S2. Overlap integrals and the calculated Förster distances for the donor-acceptor pairs.

donor-acceptor pair	overlap integral, J (M^{-1} cm^{-1} nm^4)	Förster distance, R_0 (Å)
525QD-Cy3	8.54×10^{15}	67
525QD-Texas Red	2.25×10^{15}	54
Cy3-Texas Red	7.73×10^{15}	51
Cy3-Cy3	5.28×10^{15}	48
Texas Red-Texas Red	4.37×10^{15}	51

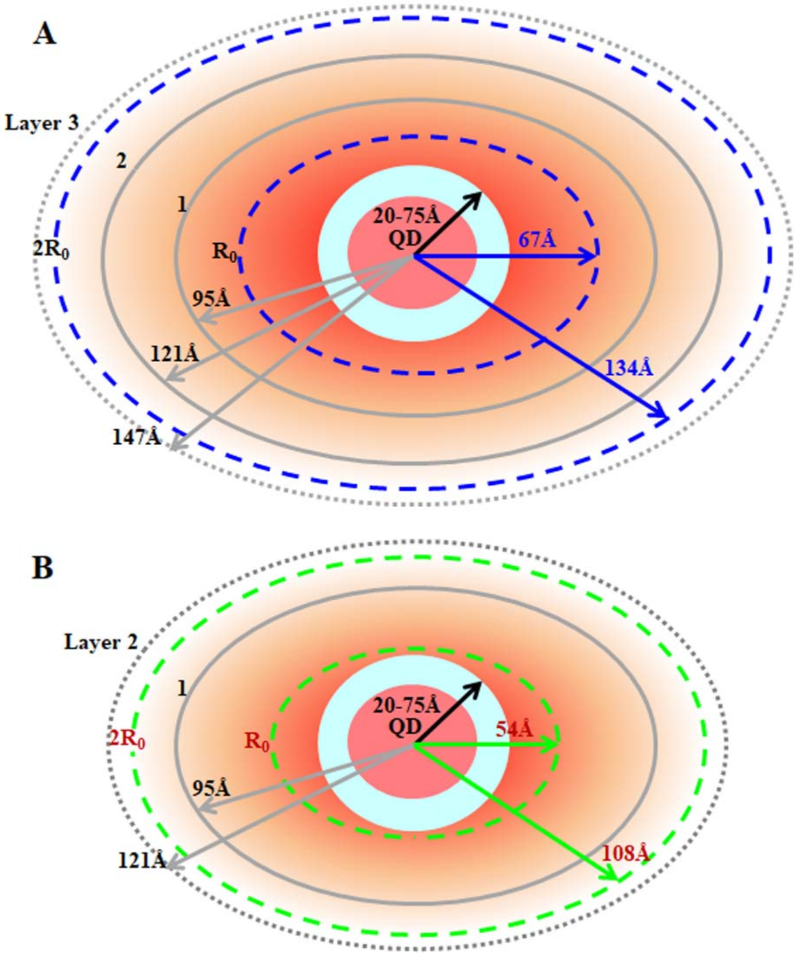

Figure S2. A schematic depicting the Förster distance (R_0) of an idealized streptavidin-functionalized 525QD donor and a Cy5 acceptor (A) or a Texas Red acceptor (B). The streptavidin-functionalized core-shell 525QD has a core radius of ~20 Å for CdSe and an overall radius of ~75 Å for streptavidin-functionalized core-shell 525QD based on the manufacturer specifications. The Förster distance (R_0) is calculated to be 67 Å for the 525QD/Cy3 pair and 54 Å for the 525QD/Texas Red pair. The distance from 525QD center to multilayer dsDNA is estimated to be 95 Å (one-layer), 121 Å (two-layer), and 147 Å (three-layer).

Study homo and hetero FRET.

For simultaneous detection of multiple miRNAs, we must ensure minimal homo-FRET and hetero-FRET interactions[1] in the QD-DNA-acceptor nanostructures. The R_0 values is 48 Å for the Cy3/Cy3 pair and 51 Å for the Texas Red/Texas Red pair (Table S2). The HRCA reaction involves a 70~71-nt circular template (Table 1) and produces a long repeat sequence, with each repeat being bound by a reporter probe. The dye-dye distance is calculated to be ~238 Å for the Cy3/Cy3 pair and ~241 Å for the Texas Red/Texas Red pair,[2] far beyond the efficient distance of $2R_0$ (R_0 = 48 Å for the Cy3/Cy3 pair, R_0 = 51 Å for the Texas Red/Texas Red pair). Thus, the homo-FRET between the dyes is negligible.[1] Notably, even though there is an overlapped region between the emission spectrum of Cy3 and the absorption spectrum of Texas Red (Figure S1 and Tables S1 - S2, R_0 = 51 Å for the Cy3/Texas Red pair), no FERT occurs between Cy3 and Texas Red because the Cy3-labeled reporter probe cannot be linked to the Texas Red-labeled reporter probe.

Both the stacked homo-FRET and hetero-FRET are negligible because (1) the addition of reporter probe to the 525QD at the ratio of 15:1 is much less than the maximum number of repeats which may be allowed to assemble on the surface of single QD (i.e., maximum 111 Cy3 acceptors and 46 Texas Red acceptors may be assembled on the surface of single 525QD). (2) the fluorescence emission spectra of nanostructures were monitored at the excitation wavelength of 405 nm. There is negligible excitation of Cy3 and Texas Red at the excitation wavelength of 405 nm.

To investigate the homo-FRET and hetero-FRET resulting from the assembly of multilayer dsDNA onto a single 525QD, we measure the fluorescence spectra of miRNA-triggered HRCA in the absence and presence of 525QD at the excitation wavelength of 405 nm and 532 nm, respectively (Figure S3). The products of HRCA reaction can bind multiple capture probes and acceptor-labeled report probes. The subsequent addition of streptavidin-coated 525QD enables the formation of the 525QD-DNA-acceptor nanostructures via streptavidin-biotin interaction, enabling efficient FRET between the 525QD and the acceptors. However, in the

absence of streptavidin-coated 525QD, no 525QD-DNA-acceptor nanostructure can be formed and no FRET between the 525QD and the acceptors can occur. Only the assembly of multilayer dsDNA onto a single 525QD leads to the existence of stacked distances between Cy3/Cy3, Texas Red/Texas Red, and Cy3/Texas Red in the 525QD-DNA-acceptor nanostructure. The 525QD can be excited by 405 nm, but there is negligible excitation of Cy3 and Texas Red at the excitation wavelength of 405 nm. In contrast, Cy3 and Texas Red can be excited by 532 nm, but there is negligible excitation of 525QD at the excitation wavelength of 532 nm. Thus, the excitation wavelength of 405 nm may be used to investigate the FRET between 525QD and the acceptors, and the excitation wavelength of 532 nm may be used to investigate the FRET between the acceptors (i.e., Cy3/Cy3, Texas Red/Texas Red, and Cy3/Texas Red). Neither Cy3 nor Texas Red signal is observed in the absence of 525QD (Figures S3A, C, and E, blue line) at the excitation wavelength of 405 nm, but distinct Cy3 and Texas Red fluorescence signal are observed in the presence of 525QD (Figures S3A, C, and E, red line), indicating that the addition of streptavidin-coated 525QD enables the formation of 525QD-DNA-acceptor nanostructures via streptavidin-biotin interaction and subsequent efficient FRET between the 525QD and the acceptors.

For the 525QD-DNA-Cy3 nanostructure and the 525QD-DNA-Texas Red nanostructure, the fluorescence spectra in the absence of 525QD (Figures S3B and D, blue line) are consistent with those in the presence of 525QD (Figures S3B and D, red line) at the excitation wavelength of 532 nm, indicating negligible homo-FRET in both the 525QD-DNA-Cy3 nanostructure and the 525QD-DNA-Texas Red nanostructure. For the 525QD-DNA-Cy3/Texas Red nanostructure, the fluorescence spectrum in the absence of 525QD (Figure S3F, blue line) is consistent with that in the presence of 525QD (Figure S3F, red line) at the excitation wavelength of 532 nm, indicating negligible hetero-FRET in the 525QD-DNA-Cy3/Texas Red nanostructure.

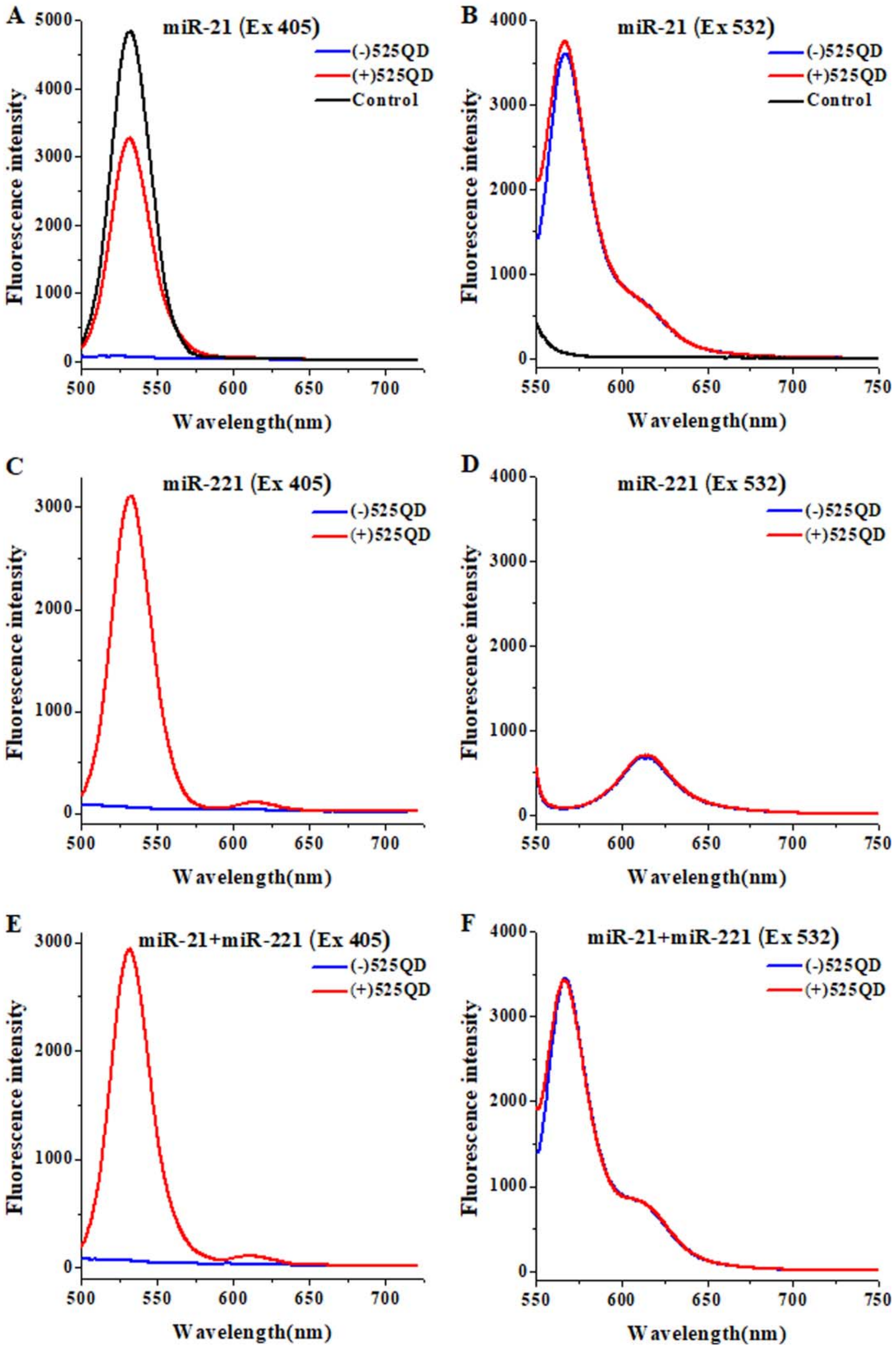

Figure S3. (A) Measurement of fluorescence spectra of miR-21-triggered HRCA using biotinylated capture probe and Cy3-labeled reporter probe in the absence (blue line) and presence of 525QD (red line) at the excitation wavelength of 405 nm. The absence of miRNA is used as the control (black line). (B) Measurement of fluorescence spectra of miR-21-triggered HRCA using biotinylated capture probe and Cy3-labeled reporter probe in the absence (blue line) and presence of 525QD (red line) at the excitation wavelength of 532 nm. The absence of miRNA is

used as the control (black line). (C) Measurement of fluorescence spectra of miR-221-triggered HRCA using biotinylated capture probe and Texas Red-labeled reporter probe in the absence (blue line) and presence of 525QD (red line) at the excitation wavelength of 405 nm. (D) Measurement of fluorescence spectra of miR-221-triggered HRCA using biotinylated capture probe and Texas Red-labeled reporter probe in the absence (blue line) and presence of 525QD (red line) at the excitation wavelength of 532 nm. (E) Measurement of fluorescence spectra of miR-21- and miR-221-triggered HRCA using biotinylated capture probe, Cy3-labeled reporter probe and Texas Red-labeled reporter probe in the absence (blue line) and presence of 525QD (red line) at the excitation wavelength of 405 nm. (F) Measurement of fluorescence spectra of miR-21- and miR-221-triggered HRCA using biotinylated capture probe, Cy3-labeled reporter probe and Texas Red-labeled reporter probe in the absence (blue line) and presence of 525QD (red line) at the excitation wavelength of 532 nm.

Study the absorption of Cy3- and Texas Red-labeled reporter probes.

We measured the fluorescence spectra in the presence of 525QD and 525QD + reporter probes, respectively (Figure S4). In the presence of target miRNA-triggered HRCA product, no fluorescence quenching of 525QDs was observed in response to Cy3-labeled reporter probes (Figure S4, red line), Texas Red-labeled reporter probes (Figure S4, blue line), and Cy3-labeled reporter probes + Texas Red-labeled reporter probes (Figure S4, magenta line), suggesting that neither Cy3 nor Texas Red fluorophore can adsorb on the surface of streptavidin-coated 525QDs.

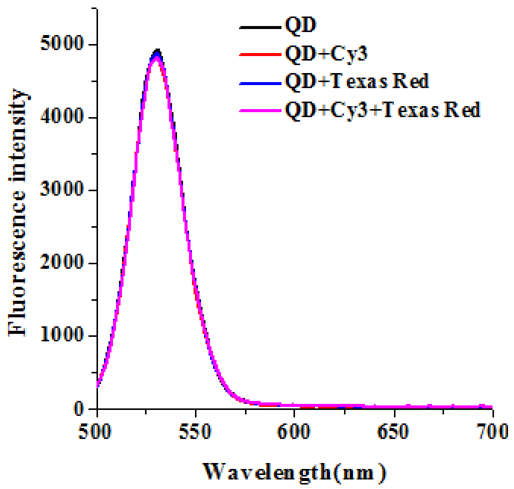

Figure S4. Measurement of fluorescence spectra of target miRNA-triggered HRCA product in the presence of 525QD, 525QD + Cy3-labeled reporter probe, 525QD + Texas Red-labeled reporter probe, and 525QD + Cy3-labeled reporter probe + Texas Red-labeled reporter probe, respectively. The excitation wavelength is 405 nm. The miR-21 concentration is 0.1 μM, and the miR-221 concentration is 0.1 μM.

Study the wrapping of multiple biotinylated HRCA product around 525QD.

We systematically investigated the wrapping of multiple biotinylated HRCA product around the 525QD via streptavidin-biotin interaction by measuring the fluorescence spectra in the presence of 525QD and 525QD + reporter probes at various amount/concentration of biotinylated capture probe, respectively (Figures S5). When the biotinylated capture probe : reporter probe : 525QD = 0: 15: 1, no fluorescence quenching of 525QDs is observed in the presence of either miR-21-triggered HRCA product + Cy3-labeled reporter probe or miR-221-triggered HRCA product + Texas Red-labeled reporter probe, suggesting that neither Cy3 nor Texas Red fluorophore can adsorb on the streptavidin-coated 525QDs. When the biotinylated capture probe : reporter probe : 525QD = 1: 15: 1 (Figure S5), the FRET efficiency is calculated to be 7.8 % for 525QD/Cy3 pair and 13.0 % for 525QD/Texas Red pair on the basis of eq 5. The average donor-acceptor separation distance was calculated to be 158 Å for 525QD/Cy3 pair and 117 Å for 525QD/Texas Red pair on the basis of eq 4. When the biotinylated capture probe :

reporter probe : 525QD = 5: 15: 1 (Figure S5), the FRET efficiency is calculated to be 24.0 % for 525QD/Cy3 pair and 25.2 % for 525QD/Texas Red pair on the basis of eq 5. The average donor-acceptor separation distance was calculated to be 128 Å for 525QD/Cy3 pair and 102 Å for 525QD/Texas Red pair on the basis of eq 4. When the biotinylated capture probe : reporter probe : 525QD = 15: 15: 1, the FRET efficiency is calculated to be 33.7 % for 525QD/Cy3 and 35.8 % for 525QD/Texas Red pair on the basis of eq 5, much higher than the values measured when the biotinylated capture probe : reporter probe : 525QD = 1: 15: 1 and 5: 15: 1. The average donor-acceptor separation distance were calculated to be 118 Å for 525QD/Cy3 pair and 93 Å for 525QD/Texas Red pair on the basis of eq 4, much shorter than the distances measured when the biotinylated capture probe : reporter probe : 525QD = 1: 15: 1 and 5: 15: 1, suggesting the wrapping of multiple biotinylated HRCA product around the 525QD via streptavidin-biotin interaction, which leads to the shorter average donor-acceptor separation distance and consequently the improved FRET efficiency. On the basis of the assumption that the radius of the streptavidin-functionalized 525QD is ~75 Å, the effective width (r1) of multilayer dsDNA is calculated to be 43 Å for the 525QD/Cy3 pair and 18 Å for the 525QD/Texas Red pair, the unhydrated helical diameter of dsDNA is 20 Å and an interhelical gap produced by electrostatic repulsion is 6 Å under the buffer conditions,[2] the number of double-helical domains along that axis (H) is calculated to be ~2 for the 525QD/Cy3 pair and ~1 for the 525QD/Texas Red pair on the basis of eq 3. i.e. two-layer dsDNA for the 525QD/Cy3 pair and one-layer dsDNA for the 525QD/Texas Red pair.

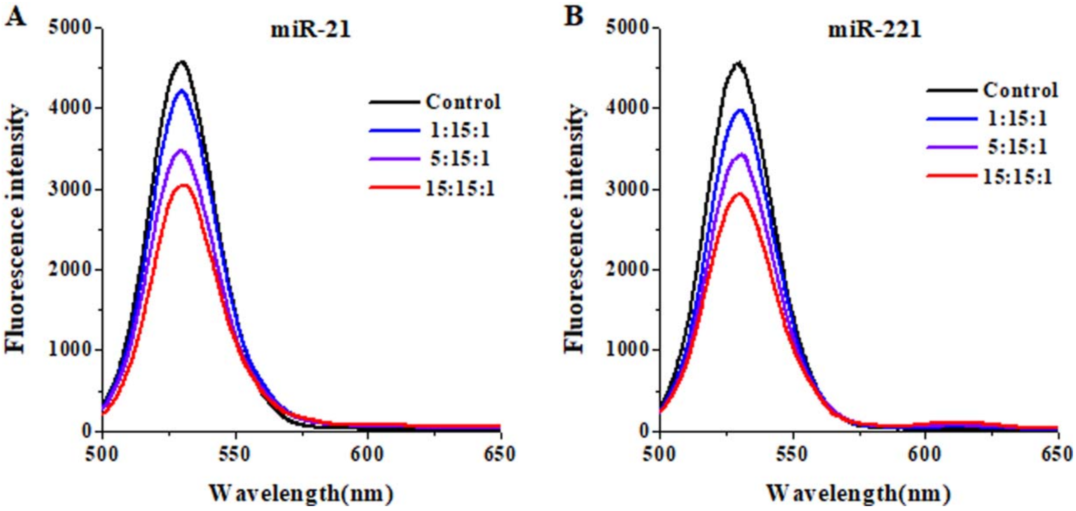

Figure S5. (A) Measurement of fluorescence spectra of HRCA product in the absence (control, black line) and presence of miR-21 with the biotinylated capture probe : Cy3-labeled reporter probe : 525QD = 1: 15: 1 (blue line), 5:15:1 (violet line) and 15:15:1 (red line) at the excitation wavelength of 405 nm. (B) Measurement of fluorescence spectra of HRCA product in the absence (control, black line) and presence of miR-221 with the biotinylated capture probe : Texas Red-labeled reporter probe : 525QD = 1: 15: 1 (blue line), 5:15:1 (violet line) and 15:15:1 (red line) at the excitation wavelength of 405 nm.

Enhanced signal induced by HRCA

To investigate the signal amplification induced by RCA (relative to input miRNA), we measured the fluorescence signal in response to only miRNA and HRCA reaction triggered by target miRNA with SYBR Gold as the fluorescent indicator (Figures S6A and B). Notably, the fluorescence signal with the involvement of HRCA is extremely higher than that without HRCA in response to same amount of target miRNA (Figures S6A and B, red lines). Near zero fluorescence signal is obtained without the involvement of HRCA despite the existence of same amount of target miRNA (Figures S6A and B, blue lines).

We further estimate the fold amplification.[3] The value of F - F_0 is used to calculated the number of repeats based on the obtained regression equation (Figures S6C and D), where F_0 and F are the fluorescence intensity at

540 nm in the absence (Figures 1B and C, red lines) and presence of target miRNAs (Figures 1B and C, blue lines), respectively. The number of repeats were estimated to be 4.86 pmol for miR-21-triggered HRCA and 6.03 pmol for miR-221-triggered HRCA according to the calibration curve in Figures S6C and D. Fold amplification was calculated by dividing the number of tandem repeats (calculated from total DNA synthesis) by the input miRNA (4 fmol of miR-21 and 4 fmol of miR-221). Fold amplification were calculated to be 1215 for miR-21-triggered HRCA and 1507 for miR-221-triggered HRCA. These results suggest that HRCA can significantly enhance the detection signal.

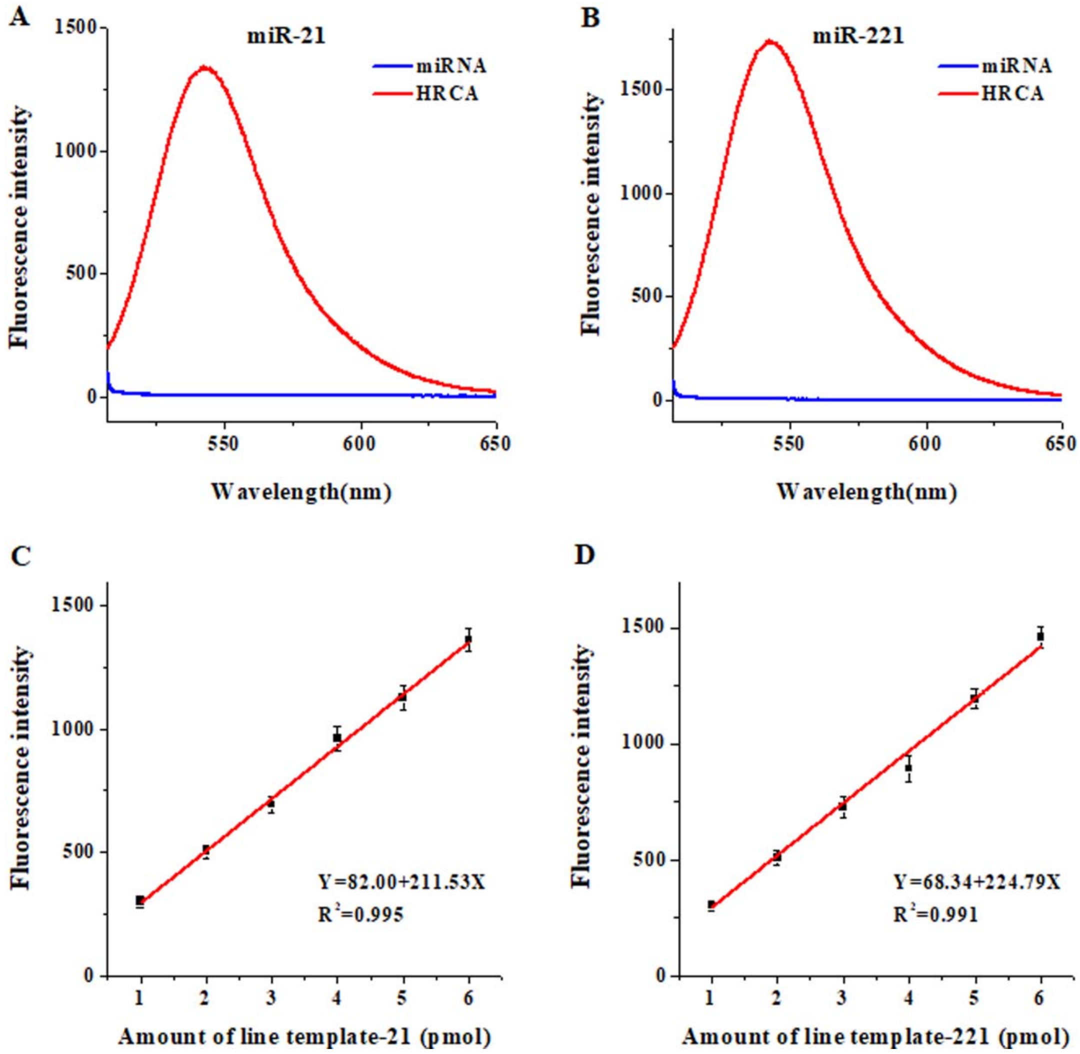

Figure S6. (A) Measurement of fluorescence spectra with SYBR Gold as the fluorescent indicator in response to only 0.2 nM miR-21 (blue line) and 0.2 nM miR-21-triggered HRCA (red line) at the excitation wavelength of 495

nm. (B) Measurement of fluorescence spectra with SYBR Gold as the fluorescent indicator in response to only 0.2 nM miR-221 (blue line) and 0.2 nM miR-221-triggered HRCA (red line) at the excitation wavelength of 495 nm. (C) Variance of fluorescence intensity as a function of the concentration of linear template-21 at the excitation wavelength of 495 nm. (D) Variance of fluorescence intensity as a function of the concentration of linear template-221 at the excitation wavelength of 495 nm. Error bars show the standard deviation of three experiments.

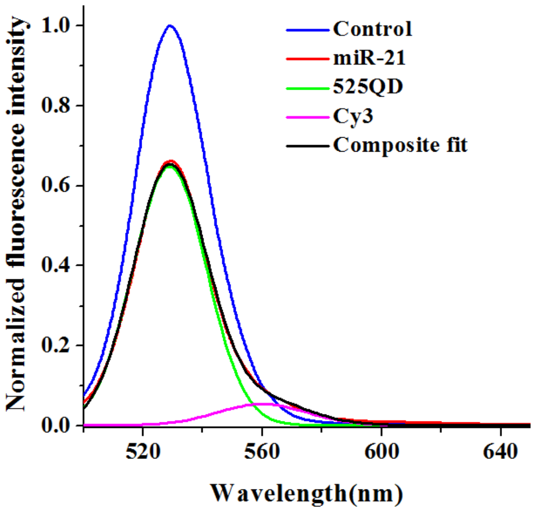

Figure S7. Measurement of 525QD and Cy3 fluorescence spectra in the absence (control, blue line) and presence of miR-21 (red line) at the excitation wavelength of 405 nm. The composite spectrum is shown in red. The fitted spectrum is shown in black, and individual contributions to the fit are shown as green (525QD) and magenta (Cy3) lines.

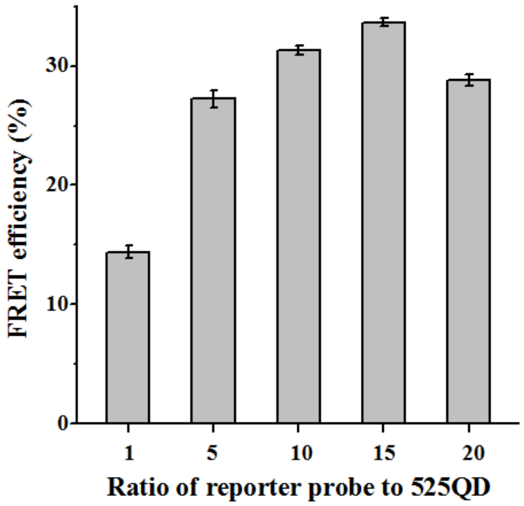

Figure S8. Variance of FRET efficiency as a function of the ratio of Cy3-labeled reporter probe to 525QD in the 525QD-Cy3 FRET system at the excitation wavelength of 405 nm. The molar ratio of biotinylated capture probe to Cy3-labeled reporter probe is kept at 1:1. Error bars show the standard deviation of three experiments.

Optimization of experimental conditions.

We performed fluorescence measurement with SYBR Gold as the fluorescent indicator to optimize the experimental conditions including the concentrations of circular template, reverse primer and dTNPs, and HRCA reaction temperature (Figure S9). We examined the influence of experimental conditions upon the value of $(F - F_0) / F_0$, where F and F_0 are the fluorescence intensity at 540 nm in the presence and absence of miR-21, respectively. The high-concentration circular template can lead to high HRCA efficiency, but it might increase the HRCA background signal correspondingly.[4] In addition, the excess circular templates may hybridize with the amplification products, biotinylated capture probes and acceptor-labeled reporter probes, preventing the formation of 525QD-DNA-acceptor complexes which are the prerequisites for FRET. On the other hand, the low-concentration circular templates can decrease the background, but it may induce low amplification efficiency due to the lack of templates. As shown in Figure S9A, the $(F - F_0) / F_0$ value improves with the increasing concentration of circular template from 0.2 to 10 nM, but it decreases beyond the concentration of 10 nM. Thus, 10 nM is selected as the

optimum concentration of circular template for HRCA reaction. As shown in Figure S9B, the $(F - F_0) / F_0$ value improves with the increasing concentration of reverse primer from 10 to 100 nM, followed by the decrease beyond the concentration of 100 nM. This can be explained by the fact that high-concentration primers might adversely cause primer dimerization and nonspecific amplification.[5] Thus, 100 nM reverse primer is used in the subsequent research. As shown in Figure S9C, the $(F - F_0) / F_0$ value improves with the increasing concentration of dNTPs from 2 to 200 μM, but it tends to saturate beyond the concentration of 200 μM. Thus, 200 μM is selected as the optimum concentration of dNTPs. As shown in Figure S9D, the $(F - F_0) / F_0$ value improves with the increasing reaction temperature from 55 to 60 °C, followed by the decrease beyond the temperature of 60 °C. This can be explained by the fact that the reaction temperature may affect the enzyme activity which is close associated with the amplification efficiency of HRCA. Thus, 60 °C is used as the reaction temperature of HRCA reaction.

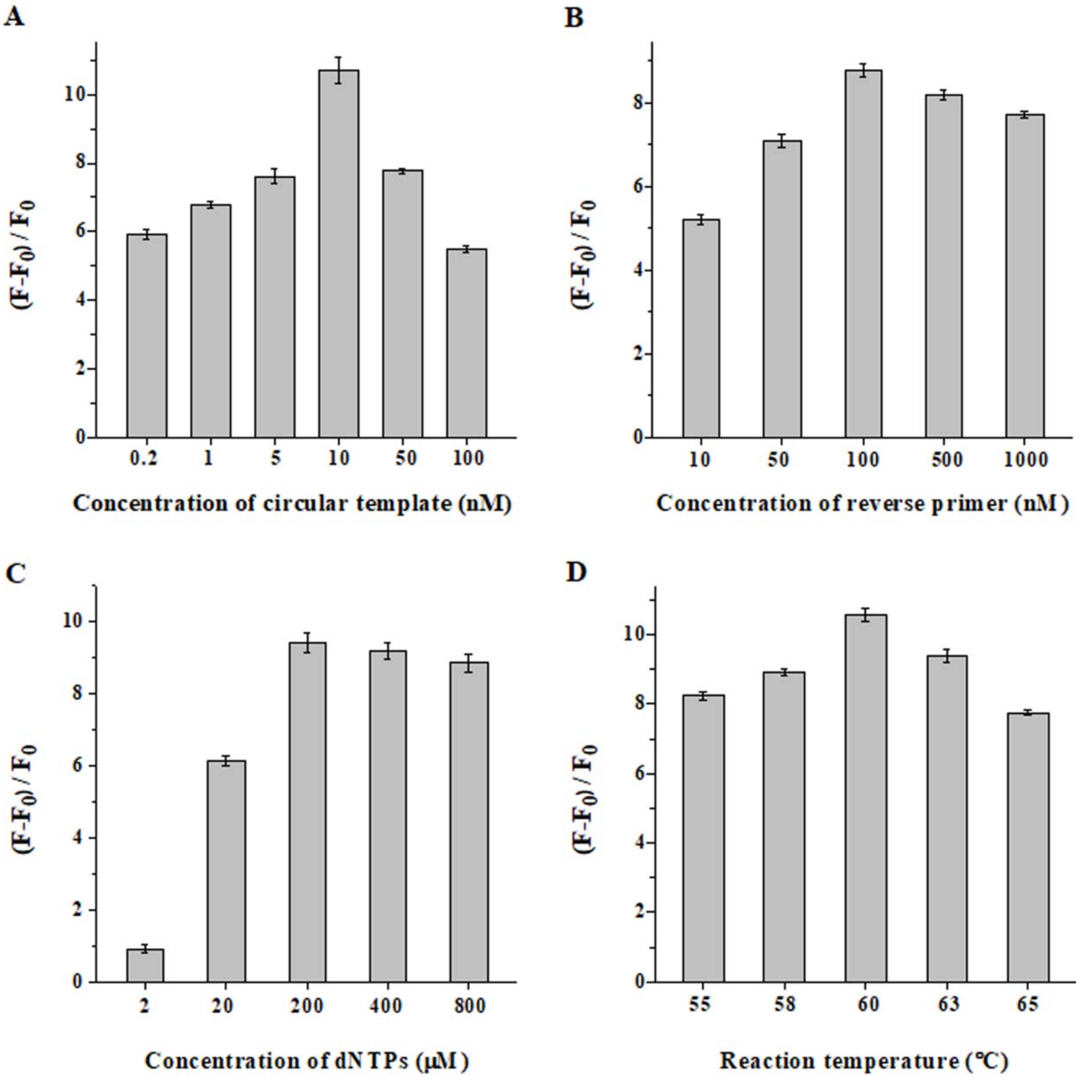

Figure S9. Variance of the $(F - F_0) / F_0$ value with the concentrations of circular template (A), reverse primer (B) and dNTPs (C), the reaction temperature (D) in the presence of miR-21 at the excitation wavelength of 495 nm. F and F_0 are the fluorescence intensity at 540 nm in the presence and absence of miR-21, respectively. The miR-21 concentration is 10 nM. Error bars show the standard deviation of three experiments.

Quantitative reverse-transcriptase polymerase chain reaction.

We employed the quantitative reverse-transcriptase polymerase chain reaction (qRT-PCR) to quantify miRNA extracted from MCF-7 cells, HEK293T cells and HeLa cells (Figure S11) using Mir-X™ miRNA qRT-PCR SYBR Kit (TaKaRa, Dalian, China). The qRT-PCR was performed in a BIO-RAD CFX connect Real-Time system (Singapore), and the concentration of miRNA was calculated according to the calibration curve in Figure S10. The specific primer of miR-21 is 5'- TAG CTT ATC AGA CTG ATG TTG A -3', and the specific primer of miR-221 is 5'- AGC TAC ATT GTC TGC TGG GTT TC -3'. The data were analyzed using the comparative C_T (threshold cycle) method.

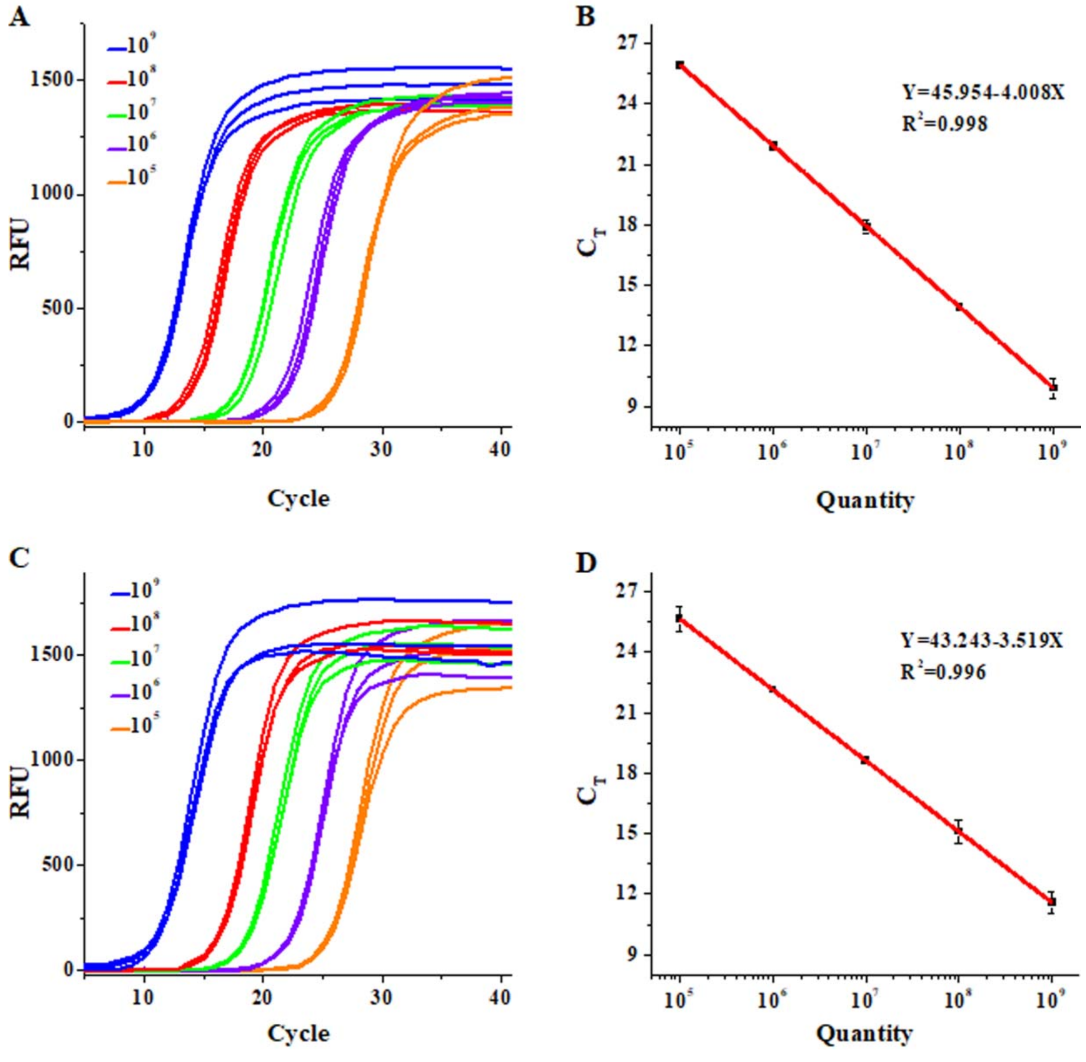

Figure S10. (A) Quantitative real-time fluorescence monitoring of miR-21 by qRT-PCR. RFU represents the

relative fluorescence units. (B) The threshold cycle (C_T) value obtained from the data (A) as a function of the quantity of miR-21. (C) Quantitative real-time fluorescence monitoring of miR-221 by qRT-PCR. RFU represents the relative fluorescence units. (D) The threshold cycle (C_T) value obtained from the data (C) as a function of the quantity of miR-221. Error bars show the standard deviation of three experiments.

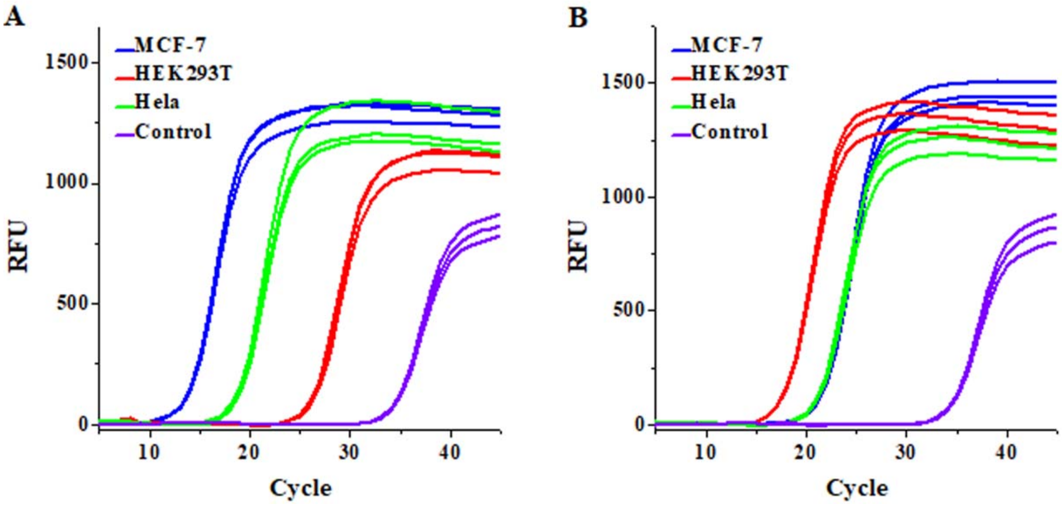

Figure S11. (A) Quantitative real-time fluorescence monitoring of miR-21 by qRT-PCR in 10 ng of total RNA extracted from MCF-7 cells, HEK293T cells, HeLa cells, and 0 ng of total RNA (control group). (B) Quantitative real-time fluorescence monitoring of miR-221 by qRT-PCR in 10 ng of total RNA extracted from MCF-7 cells, HEK293T cells, HeLa cells, and 0 ng of total RNA (control group).

References

1. K. Boeneman, D. E. Prasuhn, J. B. Blanco-Canosa, P. E. Dawson, J. S. Melinger, M. Ancona, M. H. Stewart, K. Susumu, A. Huston and I. L. Medintz, *J. Am. Chem. Soc.*, 2010, **132**, 18177-18190.
2. Y. Ke, S. M. Douglas, M. Liu, J. Sharma, A. Cheng, A. Leung, Y. Liu, W. M. Shih and H. Yan, *J. Am. Chem. Soc.*, 2009, **131**, 15903-15908.
3. G. Nallur, C. Luo, L. Fang, S. Cooley, V. Dave, J. Lambert, K. Kukanskis, S. Kingsmore, R. Lasken and B. Schweitzer, *Nucleic Acids Res.*, 2001, **29**, E118.
4. Y. Wen, Y. Xu, X. Mao, Y. Wei, H. Song, N. Chen, Q. Huang, C. Fan and D. Li, *Anal. Chem.*, 2012, **84**, 7664-7669.
5. T. Murakami, J. Sumaoka and M. Komiyama, *Nucleic Acids Res.*, 2009, **37**, e19.
